# Educational Technologies to Support Rational Antimicrobial Prescribing in Primary Healthcare: A Systematic Review

**DOI:** 10.3390/ijerph22111742

**Published:** 2025-11-18

**Authors:** Maria Karolayne de Araújo Pereira, Denise de Andrade, Ana Larissa Gomes Machado, Açucena Leal de Araújo, Priscila Rodrigues Moura de Carvalho, Maria Zélia de Araújo Madeira, Odinéa Maria Amorim Batista, Andréia Rodrigues Moura da Costa Valle

**Affiliations:** 1 Departamento de Enfermagem, Universidade Federal do Piauí (UFPI), Teresina 64049-550, PI, Brazil; analarissa2001@ufpi.edu.br (A.L.G.M.); zeliamadeira15@yahoo.com.br (M.Z.d.A.M.); oenf@ufpi.edu.br (O.M.A.B.); andreiarmcvalle@hotmail.com (A.R.M.d.C.V.); 2 Departamento de Enfermagem Geral e Especializada, Universidade de São Paulo (USP), São Paulo 05508-060, SP, Brazil; dandrade@eerp.usp.br; 3 Departamento de Enfermagem, Universidade Regional do Cariri (URCA), Iguatu 63500-000, CE, Brazil; acucena.leal@urca.br; 4 Setor de ObstetríciaMaternidade Dona Evangelina Rosa (MDER), Teresina 64014-220, PI, Brazil; priscila_moura_@hotmail.com

**Keywords:** educational technology, microbial drug resistance, primary healthcare

## Abstract

Background: Antimicrobial resistance is a global public health challenge that compromises patient safety and has gained particular relevance in primary healthcare, where the prescription of antimicrobials is frequent and often based on empirical knowledge. In this context, educational technologies emerge as strategies to strengthen professional training and promote rational antimicrobial use. Methods: A systematic review of randomized controlled trials was conducted, registered in PROSPERO (CRD42024504630) and guided by the PICOS framework. The search was carried out in 14 national and international databases, including PubMed/MEDLINE, Embase, Cochrane Library, SCOPUS, and LILACS, with no restrictions on language or publication year. Results: A total of 763 studies were identified, of which seven met the inclusion criteria and were included in the qualitative synthesis. The educational technologies reported comprised booklets, interactive seminars, workshops, training programs, online courses, and multifaceted interventions. Inter-rater agreement was substantial (κ = 0.823), although 85.7% of the studies presented a high risk of bias, mainly related to deviations from intended interventions. Conclusions: Educational technologies show potential to support appropriate antimicrobial prescribing and represent valuable tools in preventing antimicrobial resistance, although current evidence remains limited by methodological weaknesses.

## 1. Introduction

The use of medications is increasingly being treated as a consumer commodity rather than a fundamental tool for health promotion. Although appropriate use is essential for effective healthcare, misuse represents a major public health problem. Global estimates indicate that more than 50% of medications are prescribed, dispensed, or sold inappropriately, and approximately half of patients do not correctly adhere to prescribed treatments [1]. Among the most critical consequences of this scenario is antimicrobial resistance, recognized as a global threat that compromises therapeutic efficacy and leads to the emergence of resistant microorganisms, commonly known as “superbugs” [2]. Projections suggest that, by 2050, bacterial resistance could be responsible for up to 10 million deaths annually, surpassing current cancer mortality rates and generating significant clinical, social, and economic impacts [3,4].

In clinical practice, antimicrobial resistance tends to prolong hospital stays, increase healthcare costs, and increase the risk of readmissions and mortality, especially given the limited therapeutic options available [5]. This problem was further exacerbated by the COVID-19 pandemic, which saw widespread empirical and indiscriminate use of antimicrobials [5,6]. In Primary Healthcare (PHC), inappropriate prescribing is particularly concerning. Studies indicate that a high proportion of prescriptions fail to meet recommended standards regarding the drug, dosage, and duration of treatment [7]. Globally, between 2000 and 2018, antimicrobial consumption increased by 65%, especially in low- and middle-income countries, with 80% of prescriptions occurring at the primary care level [8].

As the entry point to the health system, PHC plays a crucial role in health promotion, protection, and surveillance, including the prevention of antimicrobial resistance. Effective strategies require patient safety policies, adherence to prescribing guidelines, monitoring of antimicrobial use, compliance with legislation, and education targeting health professionals [9]. Health education has increasingly relied on educational technologies, including booklets, apps, videos, games, and podcasts, which enable accessible, contextualized, and interactive learning experiences [10,11,12,13,14]. Classified as soft, soft-hard, and hard technologies, these tools have demonstrated the ability to expand knowledge and transform professional practices across diverse care contexts [15].

Given this scenario, we sought to identify the role of educational technologies aimed at health professionals in promoting the appropriate prescription of antimicrobials in PHC.

## 2. Materials and Methods

This bibliographic review adopted a systematic approach to establish the state of knowledge regarding the effectiveness of educational technologies in antimicrobial prescribing by primary healthcare (PHC) professionals. The study followed the Cochrane Collaboration recommendations [16] and the Preferred Reporting Items for Systematic Reviews and Meta-Analyses (PRISMA 2020) guidelines [17].

### 2.1. Protocol

The systematic review protocol was developed and registered on the International Prospective Register of Systematic Reviews (PROSPERO, York University) under registration number CRD42024504630. The protocol included detailed information to guide all stages of the review, ensuring transparency, avoiding duplication of efforts, and providing a reference point for future analyses [18]. The review was conducted in the period from October 2023 to February 2024.

### 2.2. Research Question and Eligibility Criteria

The research question was guided by the PICOS framework (Population, Intervention, Comparison, Outcomes, Study design) [19]: What is the effectiveness of educational technologies compared with no intervention or traditional teaching methods used by PHC professionals in antimicrobial prescribing?

Inclusion criteria: PHC professionals; randomized controlled trials (RCTs) with an intervention group using educational technologies and a control group using traditional teaching methods; publications in Portuguese, English, or Spanish; no restrictions on the year of publication.

Exclusion criteria: Cross-sectional studies, prospective or retrospective cohorts, case–control studies, case reports or series; review articles, protocols, letters to the editor, conference abstracts, opinion articles, book chapters; and studies not fully available.

### 2.3. Data Source

The literature search was conducted on 25 January 2024, across the following databases: PubMed/MEDLINE, CINAHL, Embase, LILACS, SCOPUS, and Web of Science. Additional gray literature sources included Google Scholar, the Brazilian Digital Library of Theses and Dissertations (BDTD), CAPES Theses & Dissertations Catalog, Open Grey, and ProQuest Dissertations and Theses. Searches were performed through the CAPES Journal Portal via the Federated Academic Community (CAFe) of the Federal University of Piauí (UFPI).

### 2.4. Search Strategy

A search strategy was developed from the research question and adapted to each database using the ECU model (Extraction, Conversion, Combination, Construction, Use) [20]. Controlled vocabularies (MeSH, DeCS, EMTREE) and natural language terms were combined with Boolean operators (AND, OR) to optimize sensitivity [21,22]. The search strategy underwent Peer Review of Electronic Search Strategies (PRESS) to ensure quality, consistency, and transparency [23].

### 2.5. Study Selection and Data Extraction

Two reviewers, assisted by a librarian, independently screened titles and abstracts. Duplicates were removed using the RAYYAN reference manager [24]. Disagreements on inclusion were resolved by discussion or, if necessary, by a third reviewer. Full texts of selected studies were independently read, and eligibility criteria were applied again. Inter-rater agreement was evaluated using Cohen’s Kappa coefficient (κ) with a 95% confidence interval [25,26] via Programa Statistical Package for the Social Sciences (SPSS) version 29.

Data were extracted independently using a standardized form, including study characteristics (title, author, year, country, design, objective), population, intervention details (recruitment, duration, type of technology, control group, inclusion/exclusion criteria, masking, randomization, follow-up), and outcomes (measures, main results, statistical analyses, conclusions). The PRISMA flow diagram was used to detail study selection steps.

### 2.6. Risk of Bias Assessment

Risk of bias in included RCTs was evaluated using the Cochrane Collaboration Risk of Bias Tool (RoB 2) [16], considering randomization, allocation concealment, blinding, incomplete outcome data, selective reporting, and other biases. Two reviewers independently assessed each study, with a third reviewer resolving discrepancies. Domains were classified as “low risk”, “some concerns”, or “high risk”. RevMan 5.4 was used to generate a summary graph of bias.

### 2.7. Data Synthesis

A descriptive synthesis of study characteristics was presented in tables. Due to heterogeneity in interventions and populations, a qualitative synthesis of results was conducted.

## 3. Results

Due to the heterogeneity of the included studies, particularly regarding the types of educational technologies, study populations, and outcome measures, a meta-analysis was not performed. Therefore, a qualitative synthesis was conducted to summarize the evidence.

### 3.1. Identification and Selection of Studies

**Figure 1:**
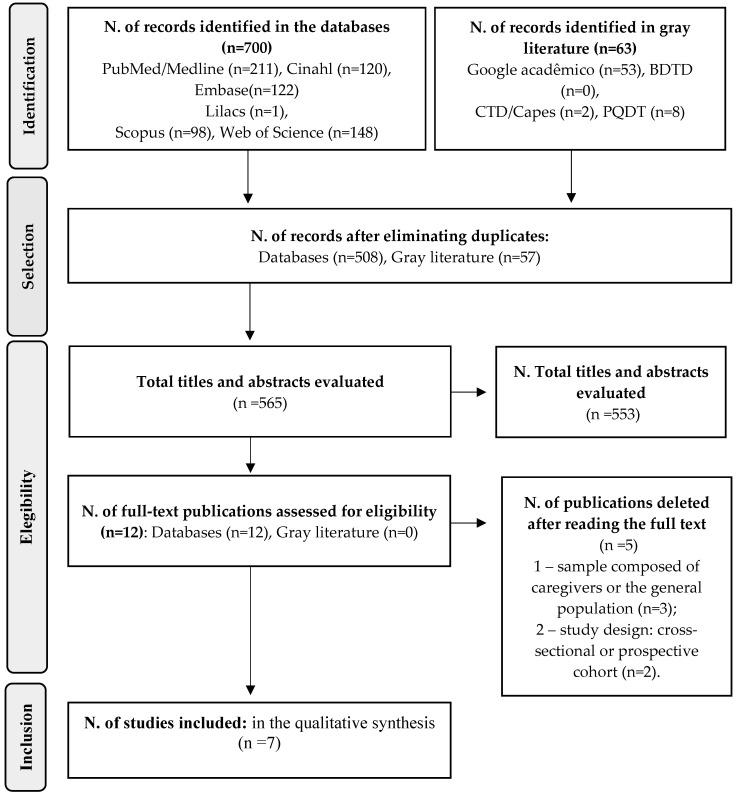
Flowchart for selection of studies for the Systematic Review. 2024.

The initial search identified 763 studies. After removing duplicates, 565 publications remained. A full screening of titles and abstracts led to the exclusion of 553 records, leaving 12 articles for full-text eligibility assessment. Manual screening of reference lists from eligible studies did not identify additional articles. During full-text evaluation, five studies were excluded, with reasons documented. Ultimately, seven articles were selected for data extraction and qualitative synthesis. The process of identification and selection is detailed in Figure 1.

### 3.2. Qualitative Synthesis of Studies

#### 3.2.1. Study Characteristics

Table S1 summarizes the characteristics of the included studies. The final sample comprised seven Randomized Controlled Trials (RCTs) conducted in Spain [27,28], California, USA [29], Wales [30], Belgium [31], Switzerland [32], and Germany [33].

The study populations consisted mainly of physicians. However, ref. [28] also included nurse prescribers and ref. [30] included dentists. Follow-up periods ranged from one to five years. All studies assessed the use of educational technologies in Primary Healthcare (PHC) as a strategy to improve and reduce antimicrobial prescriptions.

The educational interventions included online courses [27], workshops, seminars, and practical campaigns [29], mailed educational materials with in-person visits [30,31], interactive seminars with booklets [32], and multifaceted programs [33]. Technologies were classified by Merhy [15], as follows: light technologies, involving human interactions such as patient welcoming, relationship building, and promotion of autonomy; light-hard technologies, encompassing structured knowledge resources, including serialized booklets, educational videos, pamphlets, and posters; and hard technologies, comprising material elements such as technological equipment and registration forms. These technologies were implemented with the aim of reducing and qualifying antimicrobial prescriptions in PHC. Control groups received standard interventions or no technology.

Extracted data included author, year, country, study objective, sample characteristics, type and classification of educational technology, intervention and control details, main outcomes, and conclusions. Two independent reviewers conducted data extraction and categorization, with a third reviewer resolving any disagreements. Inter-rater reliability was assessed using the Kappa coefficient (κ = 0.823 [25]), indicating substantial agreement above what would be expected by chance.

#### 3.2.2. Risk of Bias Assessment of Included Studies

**Figure 2:**
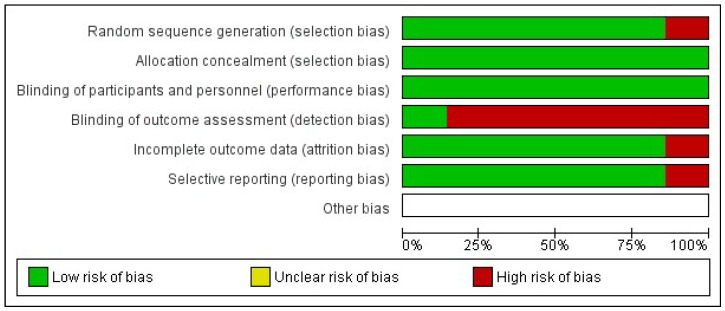
Risk of bias chart of the included studies.

**Figure 3:**
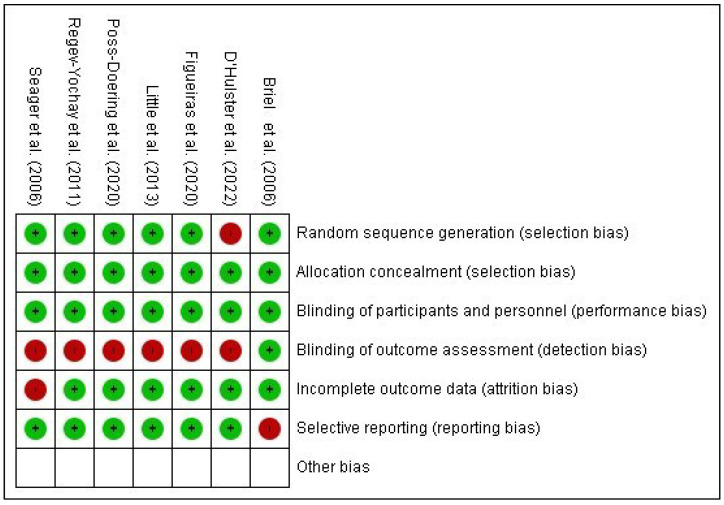
Summary of the risk of bias of the included studies Briel et al. [32], D’Hulster et al. [31], Figueiras et al. [27], Little et al. [28], Poss-Doering et al. [33], Regev-Yochay et al. [29], Seager et al. [30].

The risk of bias for included studies was assessed using the Cochrane Collaboration Risk of Bias Tool (RoB 2) for randomized trials [16] and summarized in graphs generated with RevMan 5.4 (Figure 2 and Figure 3).

One study presented low risk of bias across all domains [33]. Two studies had insufficient information to assess the “random sequence generation” domain [29,31]. One study lacked information regarding the “timing of participant identification or recruitment” [32], and another presented insufficient details for the domain “deviations from intended interventions” [29]. Six studies [27,28,29,30,31,32] exhibited a high risk of bias, particularly in the domain “deviations from intended interventions,” representing 85.7% of the included RCTs.

This integrated synthesis highlights patterns of effectiveness of educational technologies in reducing inappropriate antimicrobial prescriptions, while also identifying methodological gaps and domains of higher risk in PHC-based studies.

## 4. Discussion

This systematic review included evidence from randomized controlled trials (RCTs) assessing the effectiveness of educational technologies in antimicrobial prescribing in primary care. A total of seven studies were included [27,28,29,30,31,32,33].

The educational technologies applied in the included studies encompassed online courses [27], workshops, seminars, and practical campaigns [29], educational materials sent by mail and personal visits [30,31], interactive seminars with booklets [32], and multifaceted programs [33], compared to standard interventions (lectures or instructor-led courses where participants were passive listeners) or no intervention.

Regarding the primary outcome of reduced antimicrobial prescriptions, most studies reported positive effects on prescribing rates and/or appropriate antimicrobial use [27,29,30,31]. One study, however, presented contrasting results: the brief interactive training using an educational technology did not lower antibiotic prescription rates below common levels [32].

The study [28] expanded outcomes beyond prescription reduction, demonstrating that their evidence-based online course, grounded in national and international guidelines and utilizing videos, texts, exercises, and quizzes, effectively reduced prescription rates and associated antimicrobial resistance-related costs.

Integrating educational technologies into antimicrobial prescribing represents an innovative and effective approach to improving clinical practice, promoting responsible antibiotic use, and mitigating risks associated with antimicrobial resistance. This is particularly relevant given the global rise in antimicrobial resistance [34].

Educational technologies facilitate rapid access to updated information on antimicrobials, including prescribing guidelines, resistance patterns, and recommended treatments. Online platforms, applications, and interactive courses offer an effective way to keep healthcare professionals informed of the latest scientific evidence [35].

Among the technologies evaluated in the included studies, updated courses and training events were the most frequent. Continuing education on appropriate antimicrobial prescribing is crucial, as it equips professionals with knowledge of new agents, innovative therapeutic strategies, and the latest clinical microbiology developments, enabling evidence-informed decision making. Furthermore, addressing topics such as effective patient communication, infection prevention, and strategies to reduce unnecessary antimicrobial use contributes to a holistic and effective approach to prescribing practices [29].

Although the use of digital technologies in continuing education for prescribers offers benefits, such as knowledge gains, improved accessibility, and enhancements in motivational and cognitive factors related to clinical practice, important gaps persist. These include limited assessment of behavioral changes and clinical impact, low participant retention, and the need for strategies tailored to different professional categories, such as physicians, nurses, pharmacists, and paramedics [36,37]. Furthermore, the low adoption of these technologies by professionals may be associated with infrastructure limitations, psychological barriers, work overload, and challenges in integrating these tools into clinical routines [38].

Regarding the professional profiles in the included studies, physicians were predominant. However, one study included dentists [30], and another included prescribing nurses [28]. In primary care, nurses play a vital role in improving antimicrobial prescribing by acting as facilitators of education. They can enhance patient awareness of responsible antimicrobial use, provide guidance on adherence, emphasize the importance of completing treatment courses, and mitigate risks of self-medication. Moreover, nurses can collaborate closely with physicians and other healthcare professionals, fostering interdisciplinary dialog, reviewing prescriptions, and identifying preventive intervention opportunities [39].

In addition to physicians and nurses, pharmacists are recognized as a strategic professional group in promoting the rational use of antimicrobials [30]. They can carry out activities such as stewardship, audits, management, guideline development, and patient education. Evidence suggests that pharmacist-led interventions can improve antimicrobial prescribing and promote their rational use [40].

Potential challenges include ensuring equitable access to educational technologies across diverse healthcare settings. Successful implementation also depends on active engagement and acceptance by healthcare professionals [41]. Overall, the use of educational technologies in antimicrobial prescribing represents a valuable strategy to combat antimicrobial resistance, promoting evidence-informed clinical practices.

Training healthcare professionals in evidence-based protocols and decision support systems is essential to optimize antibiotic prescribing and, in some contexts, improve clinical outcomes compared to usual care. Evidence indicates that structured interventions incorporating digital decision support can reduce inappropriate prescriptions, enhance antimicrobial therapy, and promote safer outcomes, particularly in complex settings or units with high patient turnover [42,43,44,45,46]. The success of these initiatives therefore depends on continuous professional education, institutional support, and adequate technological infrastructure.

Future research should evaluate educational technologies across other professional categories beyond prescribers, as antimicrobial use extends beyond prescription activities [47]. Future research should explore the combination of online education, decision support, and digital technologies, rigorously evaluating the effectiveness, cost-effectiveness, and sustainability of these interventions, as well as their impact across different professional categories, since antimicrobial use extends beyond the act of prescription.

Limitations of this review relate to the generalizability of findings to different primary care contexts, given variations in healthcare systems, professional experience levels, and local policies, which may influence the reception and implementation of educational technologies. Therefore, the effectiveness of educational technologies should be interpreted considering these contextual factors.

## 5. Conclusions

In conclusion, educational technologies emerge as essential tools for addressing the challenges associated with antimicrobial prescribing. Through online platforms, interactive simulations, and visual resources, these technologies have the potential to inform and empower healthcare professionals regarding responsible prescribing practices.

By providing rapid access to up-to-date information, facilitating the understanding of complex concepts, and promoting collaboration among different professional categories, educational technologies establish themselves as valuable strategies to reduce inappropriate antimicrobial use and prevent antimicrobial resistance.

In this context, physicians, dentists, and nurses play crucial roles as agents of change in the responsible management of antimicrobials. Nurses, in particular, actively participating in educational programs, contribute to the dissemination of evidence-based prescribing guidelines, encourage safe clinical practices, and strengthen communication between healthcare professionals and patients. Acting as links between the medical team and patients, nurses guide individuals on proper antimicrobial use, reinforce treatment adherence, and enhance the effectiveness of educational interventions in promoting health.

## Data Availability

No new data were created or analyzed in this study.

## Supplementary Materials

The following supporting information can be downloaded at: https://www.mdpi.com/article/10.3390/ijerph22111742/s1. Table S1: Characteristics of the Randomized Controlled Trials included in the Systematic Review.

## Abbreviations

The following abbreviations are used in this manuscript:

CAPES	Coordenação de Aperfeiçoamento de Pessoal de Nível Superior
COVID-19	Coronavirus Disease 2019
DeCS	Descritores em Ciências da Saúde
EMTREE	Embase vocabulary
MeSH	Medical Subject Headings
PHC	Primary Healthcare
PRESS	Peer Review of Electronic Search Strategies
PRISMA	Preferred Reporting Items for Systematic Reviews and Meta-Analyses
PROSPERO	International Prospective Register of Systematic Reviews
RCTs	Randomized Controlled Trials
RevMan 5.4	Cochrane software used to generate risk of bias graphs
RoB 2	Risk of Bias Tool, version 2
SPSS	Statistical Package for the Social Sciences
