# Supplementary material for: Educational Technologies to Support Rational Antimicrobial Prescribing in Primary Healthcare: A Systematic Review
Source: Int J Environ Res Public Health. 2025 Nov 18;22(11):1742. doi: 10.3390/ijerph22111742 (PMC12652827; doi:10.3390/ijerph22111742)
Supplement: Supplementary file 1 [file ijerph-22-01742-s001.zip › ijerph-3874582 TableS1.pdf]

Table S1. Characteristics of the Randomized Controlled Trials included in the Systematic Review.

Author/Year/ Country	Objective	Sample Characteristics	Educational Technology	Intervention and Control Characteristics			Main Results	Conclusion
Figueiras/ 2020/ Espanha	Assess the effectiveness and return on investment of a multifaceted intervention.	Thirty primary care clinics in Galicia (a region in northwestern Spain) were randomly allocated into two groups: 15 received the intervention and 15 received usual care.	An online course integrated into practice accreditation and a clinical decision support system. The course was based on scientific evidence and national and international guidelines, and employed educational resources such as videos, texts, exercises, and quizzes. It was accessed through an online platform, which allowed physicians to track their progress and receive	Duration: 19 months	IG: Comprised of 1,217 physicians who received a one-hour educational visit tailored to the training needs identified in a prior study, an online course integrated into practice accreditation, and a clinical decision support system. Physicians in this group participated in the online course on the management	GC: Comprised of 1,393 physicians who did not receive any form of continuing education, guidance, or feedback on the research topic.	There was a significant reduction in overall antibiotic prescribing. Furthermore, financial analysis indicates that the intervention also resulted in cost savings and patient contributions, yielding positive financial returns relative to the investment made in the intervention.	Interventions designed based on gaps in physicians' knowledge and attitudes regarding inappropriate prescribing can improve antibiotic prescribing and generate significant direct cost savings.

Author/Year/ Country	Objective	Sample Characteristics	Educational Technology	Intervention and Control Characteristics			Main Results	Conclusion
			immediate feedback. (tecnologia dura)		of acute respiratory infections.			
Regev-Yochay/ 2011/ Califórnia	To evaluate whether the intervention among physicians and the treated population could achieve a sustained reduction in antibiotic use, specifically in classes known to promote antibiotic resistance among children in a community setting.	Fifty-two primary care pediatricians and 88,000 children registered in their practices. The intervention was led by local leaders and involved the participating physicians. It included physician focus group meetings, workshops, seminars, and practical campaigns.	Workshops, seminars, and practical campaigns. An initial 2-day interactive workshop (conducted at the beginning of Year 1) focused on identifying determinants of inappropriate antibiotic prescribing and potential interventions to reduce such prescribing. A second workshop (conducted at the beginning of Year 2) focused on	Duration: Pre-intervention = 2 years, Intervention = 3 years, Follow-up = 1 year	GC: 24 primary care pediatricians	GI: 26 primary care pediatricians	A decrease in the overall antibiotic prescribing rate (APR) among children treated by intervention physicians compared to those treated by control physicians was observed in the first year of the intervention (APR decrease among control physicians, 40%; APR decrease among intervention physicians, 22%; relative risk [RR], 0.76; 95% confidence	The multifaceted intervention involving physicians in an educational process is effective in reducing antibiotic prescribing rates and may be sustainable.

Author/Year/ Country	Objective	Sample Characteristics	Educational Technology	Intervention and Control Characteristics			Main Results	Conclusion
			communication between parents and physicians, and a third workshop (conducted at the beginning of Year 3) focused on APR feedback. (Light technology)				interval [CI], 0.75–0.78).	
Seager/ 2006/ País de Gales	To evaluate the effect of educational visits on antibiotic prescribing for acute dental pain in primary care.	General dentists were randomly recruited and assigned to one of the three study groups. Initially, 97 dentists agreed to participate in the study; however, after randomization, 27 professionals withdrew.	Educational materials sent by mail and an academic detailing visit by a trained pharmacist (control group, guidance group, or intervention group). (Light–hard technology)	Duration: -	GC: 32 with no intervention	GI: Guidance – 32 and academic detailing = 33	Patients in the intervention group received significantly fewer antibiotic prescriptions than patients in the control group (OR [95% CI] 0.63 [0.41; 0.95]) and significantly fewer inappropriate antibiotic prescriptions (OR [95% CI] 0.33 [0.21; 0.54]).	Evidence-based guidelines alone do not improve prescribing by general dentists. However, educational visits by a pharmacist can be successfully used to enhance prescribing.

Author/Year/ Country	Objective	Sample Characteristics	Educational Technology	Intervention and Control Characteristics			Main Results	Conclusion
D'Hulster/ 2022/ Bélgica	To evaluate the effect of implementin g online communicati on skills training.	Overall, 299 (2.88%) GPs completed TRACE and 93 (0.90%) completed INTRO, of whom 30 completed both.	Two courses (TRACE and INTRO) were delivered online, with brochures provided (Hard technology)	Duration: 1 year from the invitation to participate to 5 months after participation	GI: 10.154 GPs	GC: 10.312 GPs	There was no effect of the national implementation of TRACE and INTRO on population-level antibiotic prescribing rates (prescription rate ratio [PRR] = 0.99 [95% CI: 0.97–1.02]). GPs who actually completed TRACE prescribed fewer antibiotics (PRR = 0.93 [95% CI: 0.90–0.95]).	GPs who completed TRACE prescribed 7% fewer antibiotics, especially during the winter.
Briel/ 2006/ Suiça	To reduce antibiotic prescribing rates for acute respiratory tract infections using a brief	345 general practitioners, of whom 45 agreed to participate within the specified period. The first 30 physicians were randomized into	A booklet containing updated guidelines adapted to local conditions. This technology was presented in a 2-hour interactive	Duration: 5 months (January–May 2004)	GI: Fifteen physicians received guidance and training from the seminar, and 15 physicians received	GC: Fifteen physicians did not receive any form of training.	There was no statistically significant difference in the rate of adherence to antibiotic prescriptions between the full	Training using a single technology did not reduce antibiotic prescribing rates below a common level.

Author/Year/ Country	Objective	Sample Characteristics	Educational Technology	Intervention and Control Characteristics			Main Results	Conclusion
	training program focused on patient communication.	two intervention groups, while the remaining 15 formed the non-randomized control group.	seminar. (Light–hard technology)		training on the guidelines only.		intervention and the limited intervention (percentage difference -2.2, 95% CI -12.2 to 7.8, probability ratio adjusted for baseline characteristics 0.86, 95% CI 0.40 to 1.93).	
Paul Little/ 2013/ Bélgica, Espanha, País de Gales, Polónia, Reino Unido, Polónia, Holanda	To evaluate whether Internet-based training methods can change prescribing practices across multiple healthcare systems.	All general practices in the study center locations were approached, and all prescribing physicians and nurses in eligible UK practices who prescribed antibiotics for respiratory tract infections were invited to participate.	Internet-based training (interactive booklet and consultation technique videos) (Light–hard technology)	Duration: 17 months	GI: CRP training (62 practices), Communication training (61 practices), CRP and Communication training (62 practices)	GC: Usual care (61 practices)	The combined intervention was associated with the greatest reduction in prescribing rate (CRP risk ratio 0.53, 95% CI 0.36–0.74, p<0.0001; improved communication 0.68, 0.50–0.89, p=0.003; combined 0.38, 0.25–0.55, p<0.0001)	Internet-based interpretation training achieved significant reductions in antibiotic prescribing for respiratory tract infections across linguistic and cultural boundaries.

Author/Year/ Country	Objective	Sample Characteristics	Educational Technology	Intervention and Control Characteristics			Main Results	Conclusion
Poss-Doering/ 2020/ Alemanha	To promote appropriate antibiotic use for uncomplicated acute infections in primary care networks in Germany.	Thirty primary care clinics were randomly allocated into two groups: 15 received the ARena intervention and 15 received usual care. Inclusion criteria were clinics treating adult patients with acute respiratory infections, having at least one physician and one medical assistant, and agreeing to participate in the study. Exclusion criteria were clinics already participating in another study on antibiotic use, planning to relocate or close,	A multifaceted program called ARena. The program had three main components: continuing medical education for physicians, consisting of an online course on the management of acute respiratory infections, focusing on appropriate antibiotic prescribing and antimicrobial resistance prevention; patient counseling; and feedback for pharmacists. (Light–hard technology)	Duration: 18 months, with 12 months for intervention implementation and 6 months for outcome assessment.	IG: Comprised of 15 primary care clinics that received the ARena intervention, which consisted of a multifaceted program to promote appropriate antibiotic use.	CG: Comprised of 15 primary care clinics that received usual care, i.e., without any specific intervention to improve antibiotic use.	The mean knowledge score of the intervention physician group increased from 66.7% to 84.6%, while the mean score of the control group remained at 67.9%. Additionally, physicians in the IG reported greater confidence in their clinical decisions, higher satisfaction with patient care, and less pressure to prescribe antibiotics. They also expressed increased awareness of the risks of	The ARena intervention was effective in improving antibiotic use in primary care, reducing consumption and increasing knowledge, attitudes, and satisfaction among the physicians, patients, and pharmacists involved.

Author/Year/ Country	Objective	Sample Characteristics	Educational Technology	Intervention and Control Characteristics			Main Results	Conclusion
		or lacking internet access.					antimicrobial resistance.	
