# Supplementary material for: Educational Technologies to Support Rational Antimicrobial Prescribing in Primary Healthcare: A Systematic Review
Source: Int J Environ Res Public Health. 2025 Nov 18;22(11):1742. doi: 10.3390/ijerph22111742 (PMC12652827; doi:10.3390/ijerph22111742)
Supplement: Supplementary file 1 [file ijerph-22-01742-s001.zip › FigureS2_Search Strategy.pdf]

(("randomized controlled trial"[Publication Type] OR "controlled clinical trial"[Publication Type] OR "randomized"[Title/Abstract] OR "placebo"[Title/Abstract] OR "clinical trials as topic"[MeSH Terms:noexp] OR ("randomly"[Title/Abstract] OR "trial"[Title])) NOT ("animals"[MeSH Terms] NOT "humans"[MeSH Terms])) AND (("anti infective agents"[MeSH Terms] OR "Antimicrobial"[Title/Abstract] OR "microbicide*"[Title/Abstract] OR "antiinfective agent*"[Title/Abstract] OR "Antibiotic Prophylaxis"[MeSH Terms] OR "Anti-Bacterial Agents"[MeSH Terms] OR "Anti-Bacterial"[Title/Abstract] OR "antibiotic*"[Title/Abstract] OR "Antimycobacterial"[Title/Abstract] OR "bactericidal"[Title/Abstract] OR "Antimicrobial Stewardship"[MeSH Terms]) AND ("educational technology"[MeSH Terms] OR "Multimedia"[MeSH Terms] OR "models, educational"[MeSH Terms] OR "educational model*"[Title/Abstract] OR "instructional model*"[Title/Abstract] OR ("educational status"[MeSH Terms] OR "education"[MeSH Terms]) OR "education*"[Title/Abstract] OR "Literacy"[Title/Abstract] OR "Training Program"[Title/Abstract] OR "Teaching"[Title/Abstract] OR "pedagog*"[Title/Abstract] OR "education"[MeSH Subheading] OR "Audiovisual Aids"[MeSH Terms] OR ("online tutorial"[Title] OR "webinar"[Title] OR "Podcast"[Title] OR "Gamification"[Title] OR "Game"[Title] OR "Whiteboards"[Title] OR "Jamboard"[Title] OR "learning management system"[Title] OR "Moodle"[Title] OR "Google Classroom"[Title] OR (("asynchronous"[All Fields] OR "asynchronously"[All Fields]) AND "learning platforms"[Title]) OR ("Online"[All Fields] AND ("collaborate"[All Fields] OR "collaborated"[All Fields] OR "collaborates"[All Fields] OR "collaborating"[All Fields] OR "collaboration"[All Fields] OR "collaborations"[All Fields] OR "collaborative"[All Fields] OR "collaborative s"[All Fields] OR "collaboratively"[All Fields] OR "collaboratives"[All Fields] OR "collaborator"[All Fields] OR "collaborators"[All Fields]) AND "space"[Title]) OR "Microsoft Teams"[Title] OR "Cloud"[Title] OR "adaptive learning algorithms"[Title] OR "voice search"[Title] OR "digital reader"[Title] OR "tablet"[Title] OR "3d printing"[Title] OR "mobile technology"[Title] OR "artificial intelligence"[Title] OR "Video"[Title] OR "Robotics"[Title] OR "Multimedia"[Title] OR "virtual reality"[Title] OR "distance learning"[Title] OR "audiovisual"[Title] OR "audiobook"[Title] OR "radio"[Title] OR "sound recording"[Title] OR "tape recording"[Title] OR "television"[Title] OR "medical illustration"[Title] OR "optical storage"[Title] OR "motion picture"[Title] OR "movie"[Title] OR "instructional material"[Title] OR "instructional technology"[Title] OR "antibiogame"[Title])) AND ("drug prescriptions"[MeSH Terms] OR "Prescription Drugs"[MeSH Terms] OR "Prescription"[Title/Abstract] OR "prescribing*"[Title/Abstract] OR "Prescription Drug Misuse"[MeSH Terms] OR ("Prescription Drug Misuse"[MeSH Terms] OR ("Prescription"[All Fields] AND "drug"[All Fields] AND "misuse"[All Fields]) OR "Prescription Drug Misuse"[All Fields] OR "nmupd"[All Fields]) OR "prescriptions"[MeSH Terms] OR "Inappropriate Prescribing"[MeSH Terms] OR "Prescription Drug Monitoring Programs"[MeSH Terms]) AND ((("primary care"[Text Word] OR "general practi*"[Text Word] OR "primary health care"[Text Word] OR "community mental health services"[MeSH Terms:noexp] OR "family practice"[MeSH Terms:noexp] OR "home care services"[MeSH Terms:noexp] OR "physicians, family"[MeSH Terms:noexp] OR "community health services"[MeSH Terms:noexp] OR "community health nursing"[MeSH Terms:noexp] OR "community pharmacy services"[MeSH Terms:noexp] OR "community health workers"[MeSH Terms:noexp] OR "preventive health services"[MeSH Terms:noexp]) AND "Medline"[Filter]) OR (("primary care"[Title/Abstract] OR "general practi*"[Title/Abstract] OR "primary health*"[Title/Abstract] OR "community mental health*"[Title/Abstract] OR "family practice"[Title/Abstract] OR "family medicine"[Title/Abstract] OR "family physician*"[Title/Abstract] OR "home care"[Title/Abstract] OR "home based"[Title/Abstract] OR "home health*"[Title/Abstract] OR "community health*"[Title/Abstract] OR "community nurs*"[Title/Abstract] OR "health visit*"[Title/Abstract] OR "community pharmac*"[Title/Abstract] OR "preventive care"[Title/Abstract] OR "prevention

program*"[Title/Abstract] OR "preventive service*"[Title/Abstract] OR "preventive health"[Title/Abstract] OR "health promotion"[Title/Abstract]) NOT "Medline"[Filter])))
