# Supplementary figures and images for: Educational Technologies to Support Rational Antimicrobial Prescribing in Primary Healthcare: A Systematic Review
Source: Int J Environ Res Public Health. 2025 Nov 18;22(11):1742. doi: 10.3390/ijerph22111742 (PMC12652827; doi:10.3390/ijerph22111742)

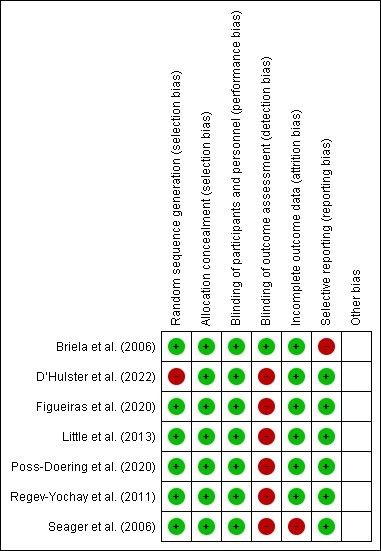

Supplement: Supplementary file 1 [file ijerph-22-01742-s001.zip › Figure_S6.jpg]

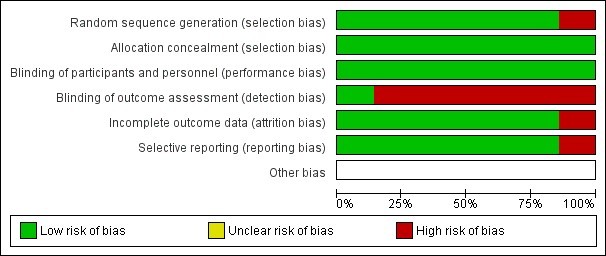

Supplement: Supplementary file 1 [file ijerph-22-01742-s001.zip › Figure_S5 (2).jpg]

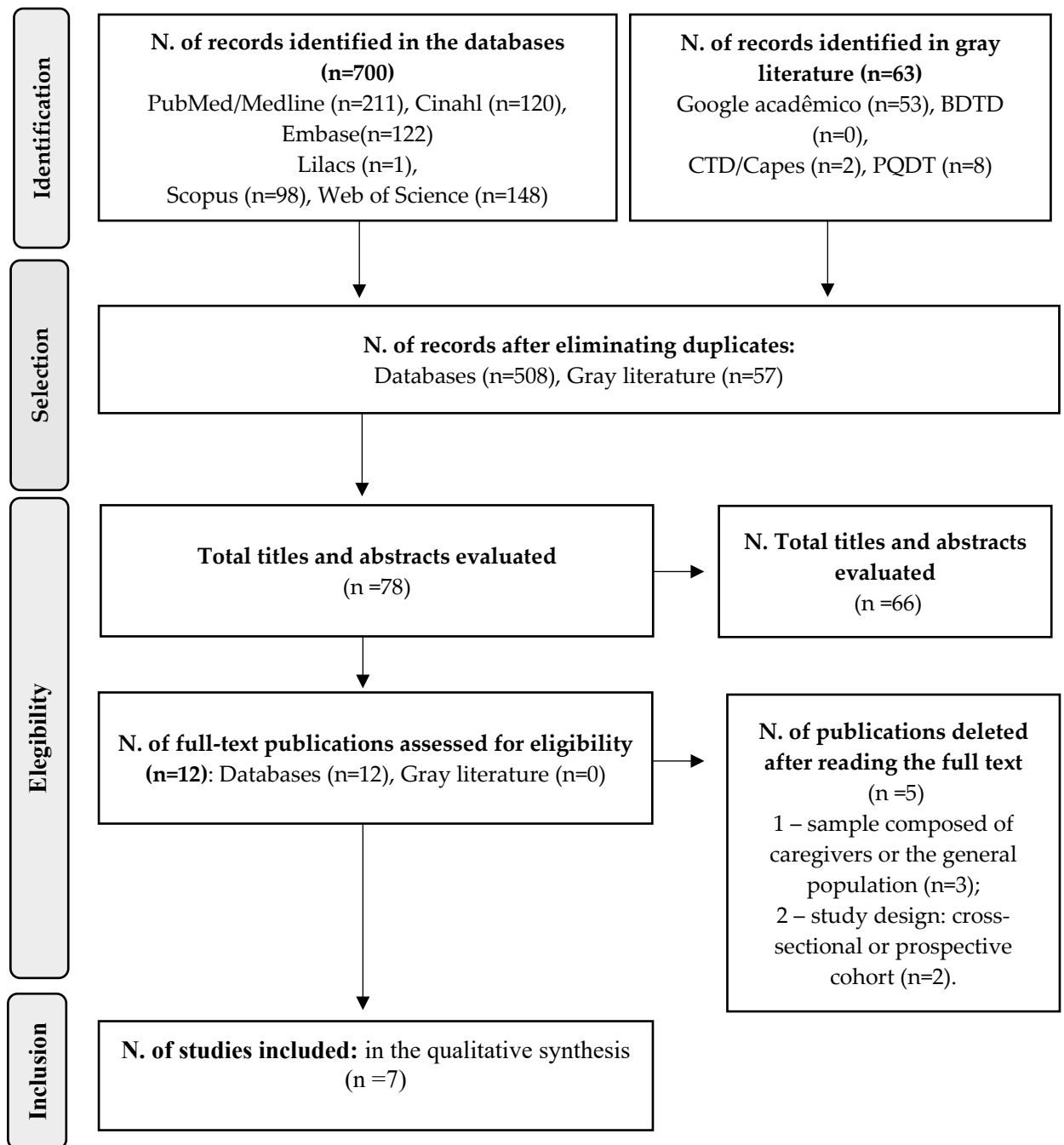

**Figure 2.** Flowchart for selection of studies for the Systematic Review. 2024.

Supplement: Supplementary file 1 [file ijerph-22-01742-s001.zip › FigureS3_Prisma_Flowchart.pdf]
